# Essentiality and centrality in protein interaction networks revisited

**DOI:** 10.1186/s12859-015-0536-x

**Published:** 2015-04-01

**Authors:** Sawsan Khuri, Stefan Wuchty

**Affiliations:** 1 Department of Computer Science, University of Miami, Coral Gables, FL 33146 USA; 2 Center for Computational Science, University of Miami, Coral Gables, FL 33146 USA

**Keywords:** PPI, Essential proteins, Protein network, Protein function

## Abstract

**Background:**

Minimum dominating sets (MDSet) of protein interaction networks allow the control of underlying protein interaction networks through their topological placement. While essential proteins are enriched in MDSets, we hypothesize that the statistical properties of biological functions of essential genes are enhanced when we focus on essential MDSet proteins (e-MDSet).

**Results:**

Here, we determined minimum dominating sets of proteins (MDSet) in interaction networks of *E. coli*, *S. cerevisiae* and *H. sapiens*, defined as subsets of proteins whereby each remaining protein can be reached by a single interaction. We compared several topological and functional parameters of essential, MDSet, and essential MDSet (e-MDSet) proteins. In particular, we observed that their topological placement allowed e-MDSet proteins to provide a positive correlation between degree and lethality, connect more protein complexes, and have a stronger impact on network resilience than essential proteins alone. In comparison to essential proteins we further found that interactions between e-MDSet proteins appeared more frequently within complexes, while interactions of e-MDSet proteins between complexes were depleted. Finally, these e-MDSet proteins classified into functional groupings that play a central role in survival and adaptability.

**Conclusions:**

The determination of e-MDSet of an organism highlights a set of proteins that enhances the enrichment signals of biological functions of essential proteins. As a consequence, we surmise that e-MDSets may provide a new method of evaluating the core proteins of an organism.

## Background

The biological importance of a protein is frequently considered a question of the number of interactions a given protein is involved in [1-3], suggesting that high topological centrality is an indicator of a protein’s importance [4-9]. In addition, such proteins are often involved in a large number of protein complexes [10], signifying that their essentiality is a consequence of their complex involvement [5,9,11-14].

Focusing on the determination of nodes that control an entire network, Liu et al. introduced a maximum matching approach to predict nodes that allowed the control of various technical, social and biological networks [15]. However, their approach was only applicable to directed networks, prompting Nacher and Akutsu to determine minimum dominating sets (MDSet) of nodes, defined as a set of centrally located nodes that provide control of undirected networks [16]. Answering the question of whether nodes that are predicted to be important for the control of interaction networks translate directly into functional sets of proteins, minimum dominating sets were found enriched with disease related and essential genes in protein interaction networks [17,18].

Here, we hypothesize that essential proteins that appear in MDSets as well (e-MDSet) enhance the enrichment signals of biological functions compared to essential proteins alone. Specifically, we considered high-quality protein interactions in *S. cerevisiae* and *H. sapiens* that have been determined by large-scale yeast two-hybrid approaches, as well as a recently released high-throughput binary interaction set in *E. coli* [19]. While highly connected proteins showed a weak enrichment of essential proteins we recovered a strong correlation between a protein’s degree and its tendency to be essential when we focused on essential proteins that appeared in the corresponding MDSet as well (e-MDSet). The impact of the combination of essentiality of a protein and its presence in the corresponding MDSet was further evidenced by our observation that e-MDSet proteins are more likely to connect protein complexes than essential proteins alone in all three organisms. Furthermore, we found that e-MDSet proteins predominantly occupied more central positions in networks and connected more protein complexes. As a corollary, e-MDSet proteins were enriched in interactions that occured within complexes, and *vice versa*. Taking a closer look at functional classifications we found that e-MDSet proteins enhance the enrichment signals of functional groups that play a role in responses to external stimuli and the physiological condition of cells.

## Results

**Figure 1:**
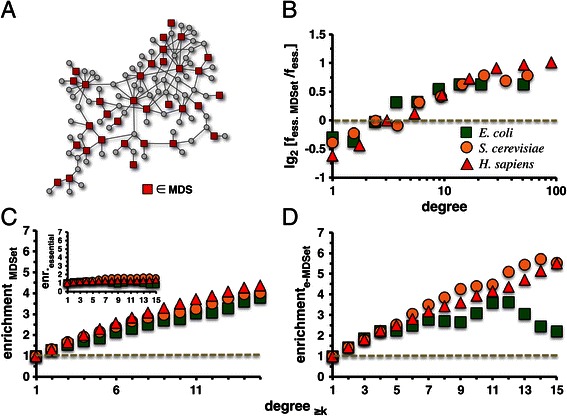
**e-MDSet proteins follow the centrality-lethality rule. (A)** In a toy network we defined a minimum dominating set (MDSet) as an optimized subset of nodes (red square symbol) from where each remaining (i.e. non-MDSet) node (gray circle symbol) can be immediately reached by one step. Therefore, each non-MDSet protein is connected to at least one MDSet protein. **(B)** After we calculated the corresponding MDSets in binary interaction networks of *E. coli*, *S. cerevisiae* and *H. sapiens*, we grouped proteins in bins of logarithmically increasing degree. In each bin we determined the fraction of essential proteins that participated in the underlying MDSet as well, allowing us to observe that essential MDSet proteins (e-MDSet) were preferably enriched among highly connected essential proteins in all organisms. In the inset of **(C)** we calculated the enrichment of essential proteins as a function of their degree in the binary protein interaction networks of *E. coli*, *S. cerevisiae* and *H. sapiens*. Generally, essential proteins in binary interactions failed to produce a significant trend in either organism. Focusing on the enrichment of MDSet proteins, however, we recovered a strong increasing trend that **(D)** was reinforced by focusing on e-MDSet proteins.

We utilized a network of 6,225 high-quality interactions between 2,640 proteins in *S. cerevisiae* and 17,523 high-quality links between 5,926 human proteins that were entirely determined by yeast-two hybrid approaches [20]. As for *E. coli*, we used a recently released, first map of 1,938 interactions between 1,203 proteins that were experimentally obtained with a yeast-two-hybrid approach [19]. As a source of information about essential genes, we collected 712 essential genes in *E. coli* and 1,110 essential genes in *S. cerevisiae* from the DEG database [21], and obtained 2,708 essential genes in *H. sapiens* from the online gene essentiality database (OGEE) [22]. We defined a MDSet as an optimized subset of proteins in an interaction network from where each remaining (*i.e.* non-MDSet) protein can be reached by one interaction. Therefore, each non-MDSet protein is connected to at least one MDSet protein (Figure 1A). In all protein interaction networks we determined corresponding minimum dominating sets by solving an integer-based linear programming problem (see Materials and Methods). In particular, we found 569 MDSet proteins in the yeast interactions network (21.26%), while there were 352 proteins in the MDSet of *E. coli* interactions (29.2%) and 940 MDSet proteins (15.9%) in the human interaction network. In comparison to all proteins (<k_all_ > = 3.9), the mean degree of MDSet proteins in the yeast interaction network was significantly increased (<k_MDSet_ > = 8.5), an observation that held for the *E. coli* (<k_all_ > = 3.2, <k_MDSet_ > = 5.6) and human networks as well (<k_all_ > = 5.3, <k_MDSet_ > = 15.5). In turn, we observed that essential genes had a slightly increased mean degree (*E. coli* < k_ess._ > = 3.4, *S. cerevisiae* < k_ess._ > = 4.8, *H. sapiens* < k_ess._ > = 6.7) compared to the corresponding values of all proteins in the underlying networks. In particular, we found 91 essential proteins that participated in the underlying MDSet proteins in *E. coli* (e-MDSet), a number that is statistically significant applying Fisher’s exact test (P = 9.2 × 10^−4^). Notably, such an enirchment pattern applied to the remaining organisms as well, where we found 179 e-MDSet proteins in *S. cerevisiae* (P = 2.3 × 10^−7^) and 209 in *H. sapiens* (P = 5.3 × 10^−3^).

### Centrality and lethality

As for a more direct comparison, we grouped proteins into bins of logarithmically increasing degree and calculated the fraction of essential genes in each group. Determining the fraction of e-MDSet proteins in each bin, Figure 1B clearly shows that e-MDSet proteins were predominantly enriched in groups of essential proteins that had an increased number of interaction partners. Considering the enrichment of essential genes as a function of the degree in the underlying interaction networks, we grouped proteins with at least a certain number of interactions and counted the number of essential proteins in each bin*.* To provide a control, we randomly sampled sets of essential genes as a null-model and defined the ratio of the observed and expected number as the enrichment of essential genes in each group. The inset of Figure 1C indicates that binary interaction networks of all organisms failed to produce a viable trend. Hypothesizing that such proteins may be enriched among highly connected proteins we repeated our initial enrichment analysis by considering MDSet and e-MDSet proteins. Indeed, we found an increasing correlation between elevated degree and their presence in MDSets in all interaction networks of *E. coli*, *S. cerevisiae* and *H. sapiens* (Figure 1C). Notably, such trends were reinforced when we focused on e-MDSet proteins, confirming our hypothesis (Figure 1D).

**Figure 2:**
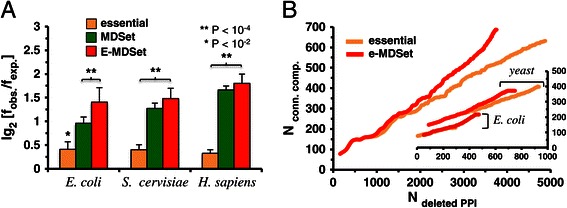
**Centrality of e-MDSet proteins. (A)** We determined the betweeness centrality of each protein in the interaction network of *E. coli, S. cerevisiae* and *H. sapiens* and chose the top 20% of proteins with highest betweeness centrality as sets of bottleneck proteins. Randomly sampling such sets 10,000 times, we observed that bottleneck proteins were weakly enriched with essential proteins in all organisms. In turn, MDSet and e-MDSet proteins were significantly enriched with bottleneck proteins. **(B)** Utilizing the subset of e-MDSet proteins in *E. coli*, *S. cerevisiae* (both in the inset) and *H. sapiens*, we sorted proteins according to their degree. Starting with the highest connected protein, we successively deleted proteins and calculated the number of connected components. To compare, we applied this procedure to a set of highest connected essential proteins of the same size in each organism. Our results suggest that the removal of e-MDSet proteins led to a lower number of deleted interactions and a higher number of connected components.

As a different measure of the central placement of essential proteins, we calculated the betweeness centrality of all proteins in the underlying networks. We defined a set of bottleneck proteins as the top 20% of proteins with highest centrality [23]. As a null-model, we randomly picked essential proteins 10,000 times and determined the enrichment of essential proteins in the corresponding sets of bottleneck proteins. Figure 2A indicates that essential proteins were weakly enriched among bottleneck proteins. While MDSet proteins were strongly enriched among bottleneck proteins in all organisms, we observed a reinforcement of these trends when we considered e-MDSet proteins, suggesting that the topological placement of MDSet proteins enhanced the enrichment signals of essential proteins (Figure 2A).

To measure a protein’s impact on an interaction network’s resilience, we performed a robustness analysis. We sorted all e-MDSet proteins according to their degree in the interaction networks of both organisms. Starting with the most connected protein we gradually deleted proteins and calculated the number of connected components after each deletion step. In comparison, we considered sets of equal size of most connected, essential proteins. Figure 2B indicates that the successive deletion of e-MDSet proteins had a higher impact on network topology by producing more connected components while removing fewer interactions than the most connected essential proteins in *E. coli*, *S. cerevisiae* and *H. sapiens*.

### Protein complexes

**Figure 3:**
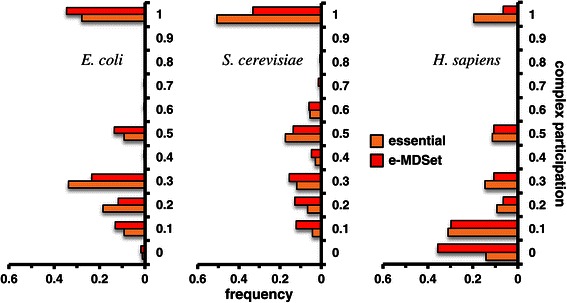
**Essential and MDSet proteins in protein complexes.** We calculated the complex participation coefficients of all proteins in the binary interaction networks of *E. coli*, *S. cerevisiae* and *H. sapiens*. Specifically, e-MDSet proteins reached into more complexes compared to essential proteins in *E. coli* (P = 1.9 × 10^−4^, Wilcoxon test), *S. cerevisiae* (P = 3.5 × 10^−6^,) and *H. sapiens* (P = 9.6 × 10^−11^).

**Figure 4:**
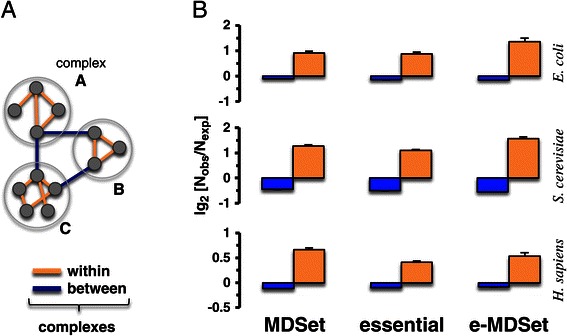
**Enrichment of interactions within and between protein complexes. (A)** Schematic illustration of interactions that appear between and within complexes. In **(B)** we determined the number of interactions between MDSet, essential or e-MDSet proteins in the same complex as well as within complexes in *E. coli*, *S. cerevisiae* and *H. sapiens*. As a random null model, we resampled proteins in complexes 10,000 times. Generally, we found that interactions between complexes appear diluted while interactions in the same complexes seemed to be enriched. While they appeared significant in sets of interactions between essential proteins we observed that such signals were enhanced in interaction sets between e-MDSet proteins (P < 10^−4^).

Moving to a higher level of cellular organization, we calculated the complex participation coefficients of proteins, a value that indicates a protein’s tendency to interact with different complexes through their interactions. The complex participation coefficient tends toward 1 if the given protein predominantly interacts with proteins in the same complex and *vice versa*. In particular, we utilized a set of 517 protein complexes in *E. coli* [24], 430 protein complexes in *S. cerevisiae* [25] and 1,843 protein complexes in *H. sapiens* [26]. Since essential proteins tend to connect more complexes than non-essential proteins, we hypothesized that the topological placement of MDSet proteins will enhance this statistical characteristic of essential proteins. The comparison of the frequency distributions of the corresponding participation coefficients in Figure 3A clearly confirmed our assumption. Indeed, e-MDSet proteins in all interaction networks largely reached into a higher number of different protein complexes compared to all essential proteins alone. On the basis of this finding, we wondered whether interactions between essential proteins are enriched within single complexes or between complexes (Figure 4A). Specifically, we counted the number of inter- and intra-complex interactions and randomly assigned the same number of proteins to each corresponding complex 10,000 times as a random null model. Generally, we observed that interactions between essential proteins connecting complexes appeared less frequently than expected in all organisms (P < 10^−4^, Figure 4B). In turn, interactions between proteins within the same complex occurred more frequently than expected in all organisms (P < 10^−4^, Figure 4B). Focusing on interactions between MDSet proteins, we found a similar, albeit slightly weaker, signal. In turn, these trends that were largely enhanced when we considered e-MDSet proteins in all organisms (P < 10^−4^, Figure 4B).

### Functional classes

**Figure 5:**
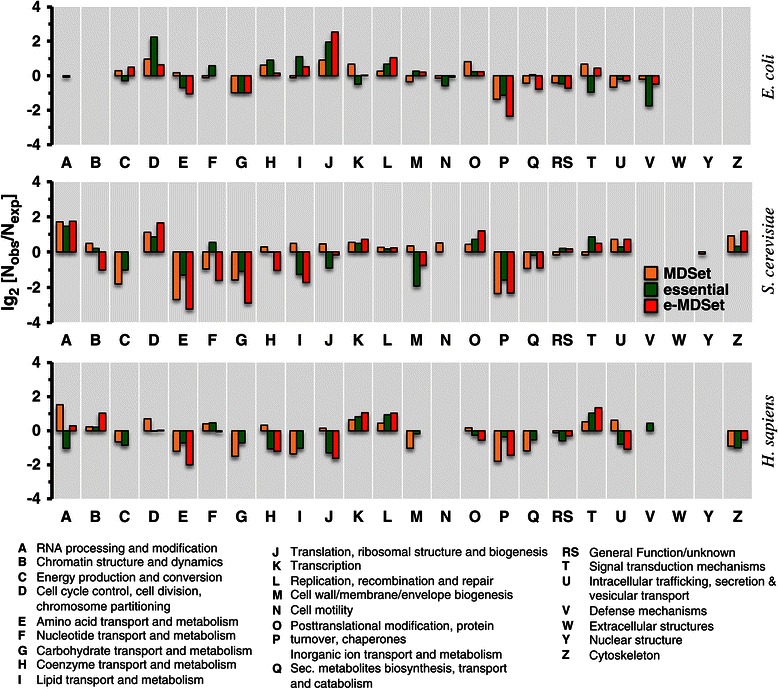
**Enrichment of functional classes.** We determined the number of proteins in the MDSet, essential or e-MDSet sets in *E. coli*, *S. cerevisiae* and *H. sapiens* that belong to the underlying functional classes. As a random null model we resampled proteins in such classes 10,000 times.

These striking network patterns are consistent with proteins that have high level regulatory functions. Therefore, we took the logical next step of grouping our protein sets according to broad functional classes that were defined by clusters of orthologous groups (COGs) [27,28]. Specifically, we counted the occurrence of essential, MDSet, as well as e-MDSet proteins in these groups (Figure 5) while we randomly assigned the same number of functional classes to each protein 10,000 times as a null model. On an organism specific level, essential, MDSet, and e-MDSets proteins roughly appeared enriched/depleted in the same functional groups in all organisms, apart from certain striking cases while enrichment patterns in the different organisms were largely incongruent. Notably, enrichment signals of essential proteins that were involved in RNA processing (group A) cell cycle control (D), transcription (K), post-translational modification (O), intracellular transport (U) and the cytoskeleton (Z) were enhanced by considering e-MDSet proteins in *S. cerevisiae*. Conversely, e-MDSet proteins were exceedingly depleted in aminoacid (A), carbohydrate (G) and lipid (I) transport and metabolism as well as chaperone and turnover activities (P) compared to essential proteins alone. While such enrichment patterns largely differ from *E. coli* and *H. sapiens*, we found that amino acid transporations and metabolism (E) and chaperones and turnover functions (P) were depleted in all organisms. In comparison to essential proteins and MDSet proteins, e-MDSet proteins in *E. coli* enhanced the enrichment signal of proteins that play a role in translation (J). In turn, cell cycle control proteins were enriched with essential proteins (D), a signal that was mitigated by e-MDSet proteins. In *H. sapiens*, we found that e-MDSet proteins enhance the enrichment signals of essential genes with transcription (K), replication (L) and signal transduction functions, while transportation (E,H,P,U) and translation (J) functions were depleted.

## Discussion and conclusions

Here, we determined minimum domainting sets (MDSets) of proteins in the underlying binary interaction networks of *E. coli*, *S. cerevisiae* and *H. sapiens* that have been entirely determined by yeast two-hybrid approaches. We defined MDSets as the smallest group of strategically placed proteins from where each remaining protein (*i.e.* non-MDSet protein) can be immediately reached through a single interaction. Therefore, each non-MDSet protein interacts with at least one MDSet protein. In other words, a MDSet is the smallest possible set of proteins that allows us to ‘cover’ all remaining proteins in the underlying networks. We hypothesized that the topological placement of MDSet proteins may correlate well with the presence of essential proteins in the underlying protein interactions networks. Indeed, we found that MDSet proteins were enriched with essential proteins in all interaction networks. Despite an absence of a correlation between the number of interaction and a protein’s tendency to be essential, we anticipated that the ability to cover other proteins may allow us to find that highly connected MDSet proteins are increasingly essential for the survival of the organism and its adaptability [17]. Indeed, we recovered a strongly ascending correlation between a protein’s lethality and its number of interactions in all organisms when we considered e-MDSet proteins.

The ability of a MDSet to ‘cover’ all proteins in a network is not just a question of finding the most connected proteins, but necessitates the determination of the lowest number of strategically placed proteins [17]. Such a concept again highlights the idea that MDSets will capture proteins that are involved in adaptability as well as lethality. While essential and MDset proteins appear enriched in bottleneck protein sets, respectively, we found that the topological placement of e-MDSet enhanced the initial trends. Furthermore, such e-MDSet proteins predominantly connect more protein complexes than essential and MDSet proteins alone, confirming our hypothesis. As for functional aspects, our analysis revealed that e-MDSet proteins broadly fell into functional classes that are vital for survival and reproduction such as cell cycle control, trafficking, cytoskeletal, translational and posttranslational modifiying functions. These functional groupings are highly dynamic in their responses to external stimuli and the physiological conditions of cells. As such, the MDSet provides an optimized set of topologically central proteins that may be contributing to the essentiality of genes, including those necessary for continued survival through a changing environment.

Notably, our results were organism-independent and strongly suggested that MDSet proteins provide a statistical enhancement of the topological and functional characteristics of essential genes. We may think of MDSet proteins as the “vital essential” set, scoring significantly higher than essential proteins alone, enhancing their topological parameters in the underlying networks. Furthermore, from a network resilience aspect, we directly compared e-MDSets with sets of essential protein hubs of equal size. Notably, we found that the deletion of e-MDSet proteins had a higher disruptive effect on the underlying networks in both organisms than the deletion of essential hub proteins alone. Taken together our results demonstrate the topological and central relevance of proteins that are involved in MDSets as well as being essential in protein interaction networks, a characteristc that is not just a matter of highest connectivity.

Most research into protein interaction networks seeks to better understand a disease mechanism or evolutionary development question. As our data collecting techniques become more sophisticated, we can ask more intricate questions of the data being collected. We conclude that the identification of MDSet proteins in this context may be crucial in elucidating the possible roles of certain genes in a pathway where they might be causing even slight perturbations.

## Methods

### Determination of a minimum dominating set (MDSet)

We defined a set *S* ⊆ *V* of nodes in a network *G = (V, E)* as a minimum dominating set if every node *v* ∈ *V* is either an element of *S* or adjacent to an element of *S* (inset, Figure 1A). In a binary integer-programming problem we assigned a binary variable *x*_*v*_ *=1* when a protein *v* ∈ *V* that participates in interactions *E* in a protein interaction network *G = (V, E)* is an element of the MDSet, and *x*_*v*_ *=0* otherwise. The smallest set of MDSet nodes is obtained by $\min {\displaystyle \sum_{v\in V}{x}_v}$, subject to the constraint ${x}_v+{\displaystyle \sum_{w\in \varGamma (v)}{x}_w\ge 1}$ where *Γ*(*v*) was the set of interaction partners of protein *v*. Since the domination problem in graphs is NP-complete no algorithm necessarily exists that allows the determination of a minimum dominating set in arbitrary graphs in polynomial time [29]. We utilized a branch-and-bound algorithm [30] as implemented by library *lpSolve* of the R programming language to solve our binary integer-programming problem.

### Protein-protein interactions of *E. coli, S. cerevisiae* and *H. sapeins*

We collected 1,938 interactions between 1,203 proteins that were experimentally determined using a yeast-two-hybrid approach in *E. coli* [19]. As for *S. cerevisiae* we collected 6,225 high-quality interactions between 2,640 proteins that were entirely determined by large-scale yeast-two hybrid approaches from the HINT database [20], including [2,31,32]. Furthermore, we assembled a network of 17,523 high-quality interactions in *H. sapiens* between 5,926 proteins from the HINT database [20]. Specifically, this set of protein interactions has been entirely determined by large-scale yeast-two hybrid approaches, including [33-36].

### Protein complexes in *E. coli*, *S. cerevisiae* and *H. sapiens*

We utilized a set of 517 protein complexes in *E. coli* from a co-affinity purification study that was followed by mass spectrometry analyses [24]. As for *S. cerevisiae*, we utilized 430 protein complexes compiled in [25], including the SGD Macromolecular Complex GO standard [37], the CYC2008 protein complex catalogue [38] and a set of manually curated protein complexes. Furthermore, we utilized 1,843 protein complexes in *H. sapiens* from the CORUM database [26].

### Protein complex participation coefficient

For each protein that is part of at least one protein complex, we defined the protein complex participation coefficient of a protein *i* as ${P}_i=\sum_{s=1}^N{\left[\frac{n_{i,s}}{\underset{s=1}{\overset{N}{{\displaystyle \sum {n}_{i,s}}}}}\right]}^2$ where *n*_*i,s*_ is the number of links protein *i* has to proteins in complex *s* out of *N* total complexes. If a protein predominantly interacts with partners of the same complex, *P* tends to *1* and *vice versa* [39].

### Enrichment analysis as a function of degree

We binned proteins in groups *N*_≥ *k*_ where each protein had at least *k* interactions and calculated the corresponding number of essential proteins *i*, *N*_*i*,≥ *k*_. Randomly picking essential genes we defined ${E}_{i,\ge k}=\frac{N_{i,\ge k}}{N_{i,\ge k}^r}$ as the enrichment of essential proteins where *N*_*i*,≥ *k*_ was the corresponding random number of essential proteins among all *N*_≥ *k*_ proteins in the corresponding bin. After averaging *E*_*i*_ over 10,000 randomizations *E*_*i*_ *>1* pointed to an enrichment and *vice versa*, while *E*_*i*_ *~ 1* indicated a random process [40].

### Bottleneck proteins

As a global measure of a nodes centrality, we calculated its betweenness centrality, indicating a proteins appearance in shortest paths through the whole network. In particular, we defined betweeness centrality *c*_*B*_ of a protein *v* as ${c}_B(v)=\sum_{s\ne t\ne v\in V}\frac{\sigma_{st}(v)}{\sigma_{st}}$, where *σ*_*st*_ was the number of shortest paths between proteins *s* and *t* while *σ*_*st*_(*v*) was the number of shortest paths running through node *v*. We defined a set of bottleneck nodes as the top 20% of interactions with highest node betweeness centrality [23].

### Functional classes

Proteins were grouped according to broad functional classes that were defined by clusters of orthologous groups (COGs) [27,28]. COGs provide a consistent classification of bacterial and eukaryotic species based on orthologous groups.
